# Coronary artery calcium and thoracic aortic calcification on non–ECG–gated chest CT in a large community-based cohort of Chinese men and women

**DOI:** 10.3389/fcvm.2026.1890920

**Published:** 2026-08-24

**Authors:** Sang Zhou, Chengrong Zheng, Weiwei Li, Zhitao Jin

**Affiliations:** PLA Rocket Force Characteristic Medical Center, Beijing, China

**Keywords:** age, coronary artery calcification, non-ECG-gated chest CT, positivity rate thresholds, sex, thoracic aortic calcification

## Abstract

**Aims:**

Non-ECG-gated chest CT frequently identifies coronary artery calcification (CAC) and thoracic aortic calcification (TAC), but age- and sex-specific thresholds remain unclear in Chinese communities. The ACC/AHA guideline recommends statins for those with CAC ≥75th percentile1. In subgroups with CAC positivity rate ≤25%, any detectable CAC corresponds to the ≥75th percentile. We aimed to determine precise sex-specific age thresholds using single-year stratification for opportunistic cardiovascular risk stratification.

**Methods:**

A total of 42 363 participants aged 1–105 years (mean age: 56.9 ± 17.3 years; 52.0% male) who underwent non‑ECG‑gated chest CT from 2023 to 2025 were enrolled in this retrospective study. CAC and TAC were visually evaluated as binary (present/absent) variables. Single‑year stratification was applied to calculate key age thresholds corresponding to 25%, 50%, and 75% positivity rates of CAC and TAC. Distributions of isolated CAC, isolated TAC, and combined vascular calcification were compared between men and women.

**Results:**

The ages at which CAC positivity reached 25%, 50%, and 75% were 48, 57, and 70 years for men versus 58, 67, and 76 years for women. Corresponding TAC threshold ages were 57, 60, and 71 years in men and 60, 67, and 78 years in women; TAC showed strong age dependence with modest sex‑related differences. The inflection age for CAC positivity exceeding 25% was approximately 58 years in women, around 10 years later than that observed in men. Isolated CAC was more frequent among men, while isolated TAC predominated in women; combined calcification became the dominant phenotype with increasing age.

**Conclusion:**

In male aged <48 years and women aged <58 years, CAC detected on non-gated chest CT may warrant statin initiation, providing additional cost-free opportunistic risk stratification beyond current guideline recommendations. Moreover, CAC-negative individuals aged <70 years in men and <76 years in women are likely to have a favorable cardiovascular prognosis.

## Introduction

Cardiovascular disease (CVD) is one of the major causes of death. Coronary artery calcification (CAC) is a marker of coronary atherosclerosis and a predictor of adverse CVD clinical outcome. For asymptomatic young adults, effective early warning tools remain limited. CAC detected on routine chest CT represents a powerful yet underutilized predictive marker for potential CVD risk in asymptomatic adults. Thoracic aortic calcification (TAC) is also frequently identified on chest computed tomography (CT) and reflects aortic atherosclerotic burden. Both CAC and TAC share traditional cardiovascular risk factors, including aging, hypertension, diabetes mellitus, dyslipidemia, and cigarette smoking. However, emerging evidence indicates that CAC and TAC may exhibit distinct epidemiological patterns and divergent prognostic implications. Notably, some individuals present with isolated CAC or isolated TAC in the absence of others. These distinct phenotypes may reflect divergent pathophysiological mechanisms: isolated CAC may indicate localized coronary atherosclerosis, whereas isolated TAC may represent more generalized age-related degenerative vascular changes. However, large-scale community-based data from Chinese populations imaged with non-ECG-gated chest CT remain scarce. Most previous studies adopted CAC scoring or ECG-gated acquisition, which entail additional radiation exposure and are not feasible for the general population in routine clinical practice.

The ACC/AHA guideline recommends statins for those with CAC ≥75th percentile regardless of age (1). Nevertheless, this criterion has not been widely adopted in clinical practice, particularly in young asymptomatic populations who seldom receive dedicated CAC testing. Here we present a simple and cost-effective approach using routine non-gated chest CT performed for non-cardiac reasons (e.g., respiratory diseases) in a large outpatient cohort, which included numerous young individuals. We determined sex-specific CAC positivity rates for each single year of age and defined the age at which the population CAC positivity rate reached 25%. Among individuals younger than this age, any detectable CAC corresponds to a CAC score ≥75th percentile. This strategy enables early identification of high-risk young asymptomatic individuals without additional radiation, allowing timely initiation of statin treatment.

## Methods

### Study population

Consecutive patients who underwent non-ECG-gated non-contrast chest CT in PLA Rocket Force Characteristic Medical Center between January 2023 and December 2025 were initially enrolled. All participants were self-referred outpatients undergoing imaging for non-cardiac indications. A total of 61,805 individuals were initially assessed. Of these, 19,442 individuals with repeated CT examinations were excluded (only the earliest scan was retained). We further excluded 307 patients with prior coronary revascularization (coronary stenting or coronary artery bypass grafting). After applying all exclusion criteria, 42,363 participants were included in the final analysis. The study protocol was approved by the Institutional Review Board of PLA Rocket Force Characteristic Medical Center (No. LC2026008). Written informed consent was waived due to the anonymized retrospective nature of the study.

### Non-ECG-gated non-contrast chest computed tomography image assessment protocol

All scans were acquired using 256-slice or higher-row CT scanners with 2.5-mm reconstruction. Two radiologists blinded to clinical data graded CAC and TAC as binary variables (present or absent). Intra- and inter-reader reproducibility was evaluated using Cohen's *κ* coefficient; intra-reader *κ* = 0.86 for CAC, 0.84 for TAC; inter-reader *κ* = 0.81 for CAC, 0.79 for TAC, indicating substantial agreement. Discrepancies were resolved by a senior radiologist. CAC was defined as hyperattenuation ≥130 HU involving an area ≥1 mm² in any coronary artery segment. TAC was defined as hyperattenuation ≥130 HU in the ascending aorta, aortic arch, or descending thoracic aorta.

### Statistical analysis

All statistical analyses were performed using IBM SPSS Statistics 24.0. A two-sided P-value < 0.05 was considered statistically significant. Continuous variables are presented as mean ± standard deviation (SD). Categorical variables are reported as frequencies and percentages [n(%)]. Between-group differences in continuous variables (CAC-positive vs. CAC-negative groups) were compared using independent-samples t-tests. Categorical variables were analyzed using Pearson *χ*² tests. Linear-by-linear association *χ*² tests were used to evaluate trends in CAC and TAC positivity rate across age groups. The likelihood-ratio test was used to confirm robustness when the expected frequency in any cell was <5 (and represented < 10% of all cells). Stratified analyses were performed according to sex, age group, CAC status (positive/negative), TAC status (positive/negative), and calcification phenotype (isolated CAC, isolated TAC, combined calcification) to characterize age- and sex-specific positivity rate patterns.

## Results

### Baseline characteristics

**Figure 1:**
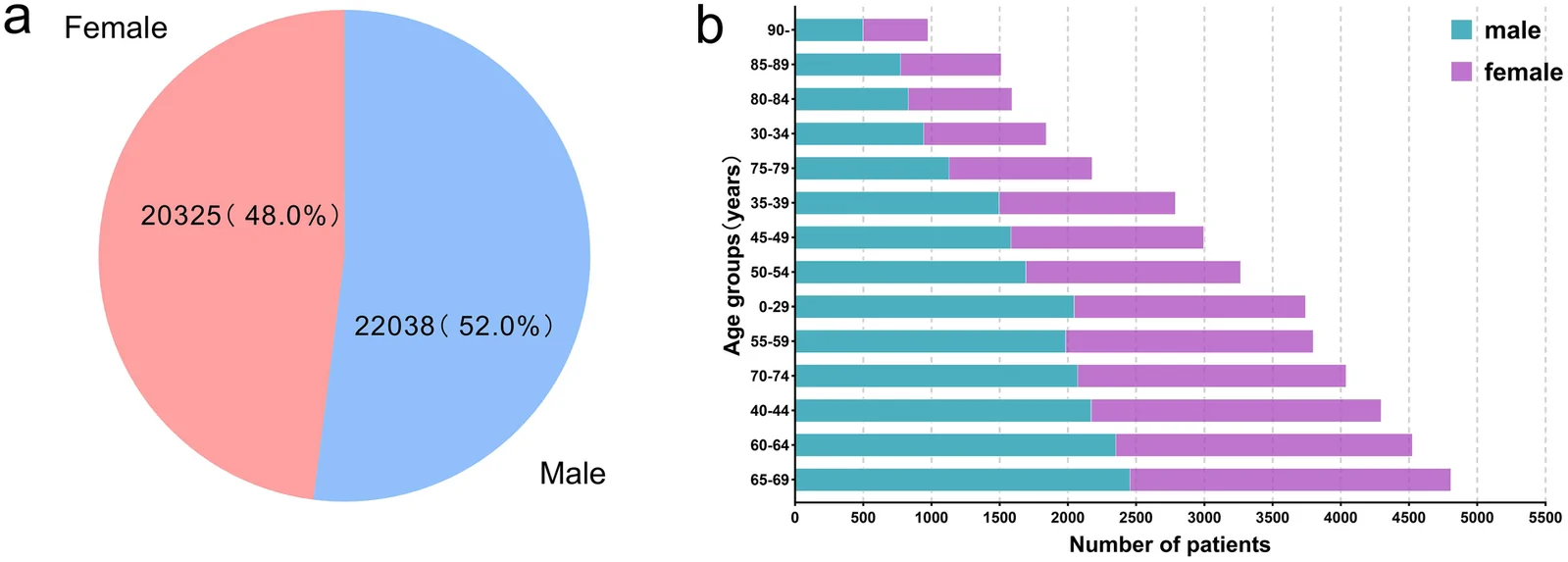
Baseline characteristics of the study cohort. **(a)** Sex distribution of the 42,363 participants. **(b)** Age and sex stratification of the study cohort.

The study cohort consisted of 42,363 community-derived participants, including 22,038 men (52.0%) and 20,325 women (48.0%), with a mean age of 56.9 ± 17.3 years (range 1–105 years). All participants were outpatients undergoing non-ECG-gated chest computed tomography (CT) for non-cardiac indications. The sex distribution and age–sex stratification of the study cohort are presented in Figure 1. Age-stratified analysis demonstrated that the study population was representative of the general community without selection bias. Specifically, there were 7,796 men aged ≤45 years and 8,553 women aged ≤55 years, collectively accounting for 38.6% of the total cohort. In addition, participants aged ≤65 years numbered 28,140, representing 66.4% of the entire study population. These results indicate that non-ECG-gated chest CT is widely utilized among young and middle-aged adults, thereby offering a valuable opportunity for the early detection of subclinical coronary artery calcification (CAC) and thoracic aortic calcification (TAC) in this at-risk population. Further elucidation of the prognostic implications of CAC and TAC may facilitate the implementation of earlier preventive strategies against coronary artery disease in young and middle-aged high-risk individuals.

### Age- and sex-specific positivity rate of CAC and TAC

Age- and sex-specific positivity rates of CAC and TAC are summarized in Table 1. Participants aged 30–89 years were categorized into 5-year age intervals for detailed trend analysis. Those aged <30 years were grouped together, as positivity rates of both CAC and TAC were extremely low in this young population, with negligible variation across single years. Conversely, individuals aged ≥90 years were combined into a single group because CAC and TAC were highly prevalent at this advanced age, with little additional stratification value.

**Table 1:** Age- and sex-specific positivity rate of CAC and TAC.

Age (y)	Male (n)	CAC, *n* (%)	TAC, *n* (%)	Female (n)	CAC, *n* (%)	TAC, *n* (%)	Total (n)
≤29	2,465	3 (0.1)	0 (0.0)	1,277	1 (0.0)	1 (0.1)	3,742
30–34	1,092	14 (1.3)	2 (0.2)	750	0 (0.0)	0 (0.0)	1,842
35–39	1,427	75 (5.3)	21 (1.5)	1,362	8 (0.6)	5 (0.4)	2,789
40–44	2,425	266 (11.0)	90 (3.7)	1,871	34 (1.8)	17 (0.9)	4,296
45–49	1,636	386 (23.6)	212 (13.0)	1,361	72 (5.3)	44 (3.2)	2,997
50–54	1,720	640 (37.2)	447 (26.0)	1,548	169 (10.9)	132 (8.5)	3,268
55–59	1,883	942 (50.0)	777 (41.3)	1,916	437 (22.8)	387 (20.2)	3,799
60–64	2,262	1,345 (59.5)	1,228 (54.3)	2,263	777 (34.3)	735 (32.5)	4,525
65–69	2,296	1,619 (70.5)	1,554 (67.7)	2,512	1,297 (51.6)	1,268 (50.5)	4,808
70–74	1,970	1,540 (78.2)	1,514 (76.9)	2,070	1,280 (61.8)	1,303 (62.9)	4,040
75–79	1,078	891 (82.7)	878 (81.4)	1,100	847 (77.0)	848 (77.1)	2,178
80–84	734	643 (87.6)	634 (86.4)	857	724 (84.5)	722 (84.2)	1,591
85–89	613	554 (90.4)	544 (88.7)	899	823 (91.5)	816 (90.8)	1,512
≥90	437	416 (95.2)	409 (93.6)	539	513 (95.2)	505 (93.7)	976
Total	22,038	9,334 (42.4)	8,310 (37.7)	20,325	6,982 (34.4)	6,783 (33.4)	42,363

Data are presented as number (percentage). Participants aged 30–89 years were categorized into 5-year age intervals; those aged <30 years were grouped together due to extremely low calcification positivity rate, and those aged ≥90 years were combined because of near-universal calcification. CAC, coronary artery calcification; TAC, thoracic aortic calcification.

**Figure 2:**
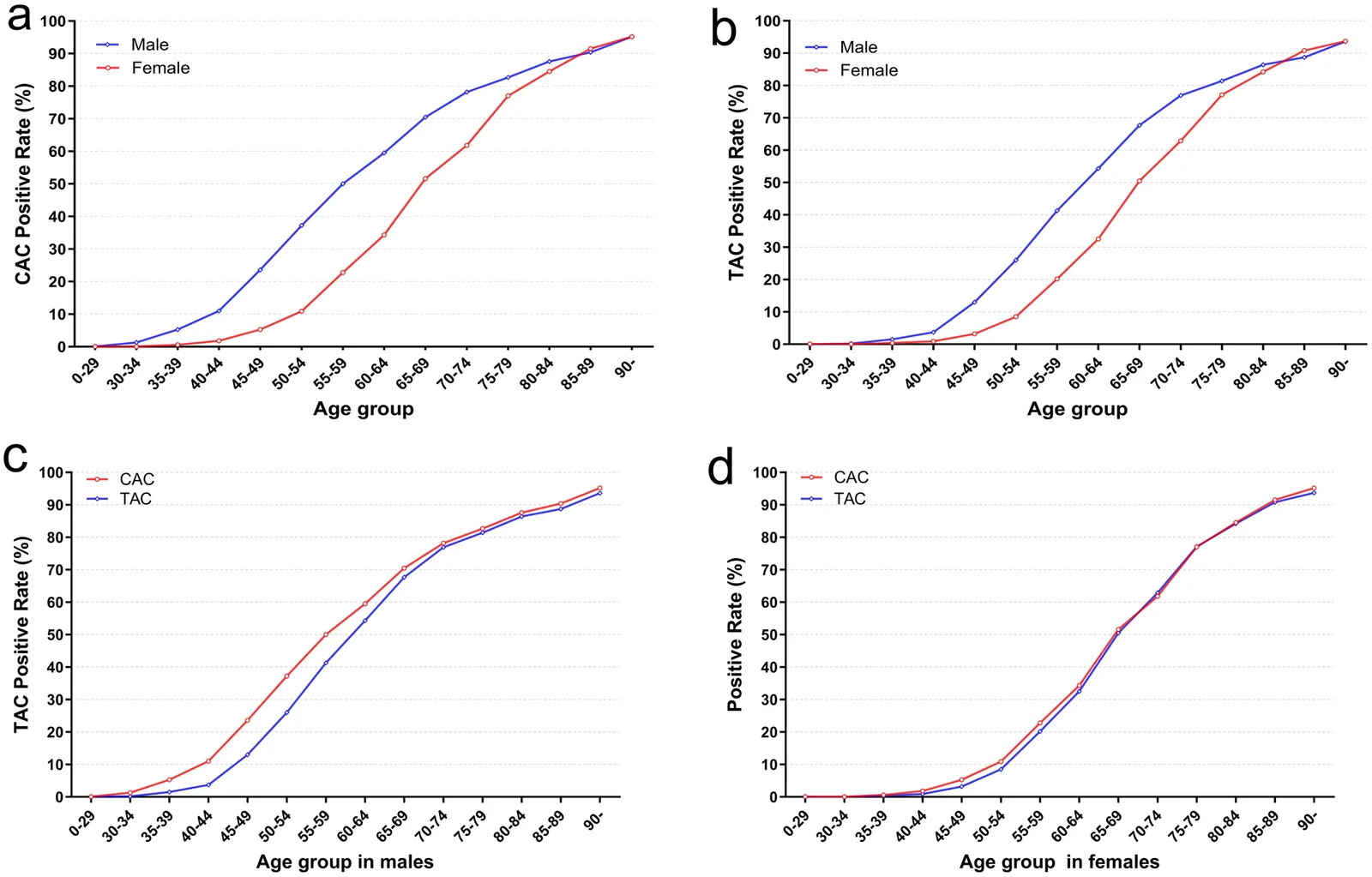
Age- and sex-specific positivity rates of coronary artery calcification (CAC) and thoracic aortic calcification (TAC). **(a)** Age-stratified positivity rates of CAC in men and women. **(b)** Age-stratified positivity rates of TAC in men and women. **(c)** Comparison of CAC and TAC positivity rates across age groups in men. **(d)** Comparison of CAC and TAC positivity rates across age groups in women. Positivity rates of CAC and TAC increased significantly with age in both sexes (all *P* for trend <0.001, tested by linear-by-linear association *χ*² test). Notably, CAC and TAC curves were nearly superimposable in women, whereas they diverged markedly in men, with TAC positivity rising more steeply than CAC positivity.

Positivity rates of both CAC and TAC increased progressively with advancing age in both men and women (all *P* for trend <0.001). As illustrated in Figures 2a,b, positivity rates of CAC and TAC were consistently and significantly higher in men than in women across nearly all age groups. In men, positivity rates remained low before age 50 years, while in women they remained low before age 55 years, followed by a steep, accelerated increase. By age 80 years, both CAC and TAC positivity rates approached 100% in both sexes.

A striking sex difference was observed in the relative progression of CAC versus TAC. In men, the TAC positivity rate rose significantly faster than the CAC positivity rate across most age groups, particularly in middle-aged and older individuals (Figure 2c). In women, by contrast, CAC and TAC positivity rates remained nearly superimposable throughout the adult lifespan (Figure 2d). The two curves diverged only minimally before age 70 years and remained tightly aligned thereafter, indicating a synchronized progression of coronary and thoracic aortic calcification in women.

Overall, the age-related increase in positivity rates was significantly steeper in men than in women for both CAC and TAC, with the most pronounced sex differences observed during middle adulthood (40–69 years).

### Age stratification of CAC and TAC positivity rate

To further refine cardiovascular risk stratification, we defined three clinically relevant positivity rate thresholds (25%, 50%, and 75%). Because conventional 5-year age grouping lacked precision, we used single-year stratification among participants aged 30–90 years to determine exact age cutoffs. Calcification was extremely rare before age 30 years, with only sporadic early-onset cases observed (see Table 1 footnote). These rare cases did not change the overall prevalence pattern in individuals younger than 30 years.

**Figure 3:**
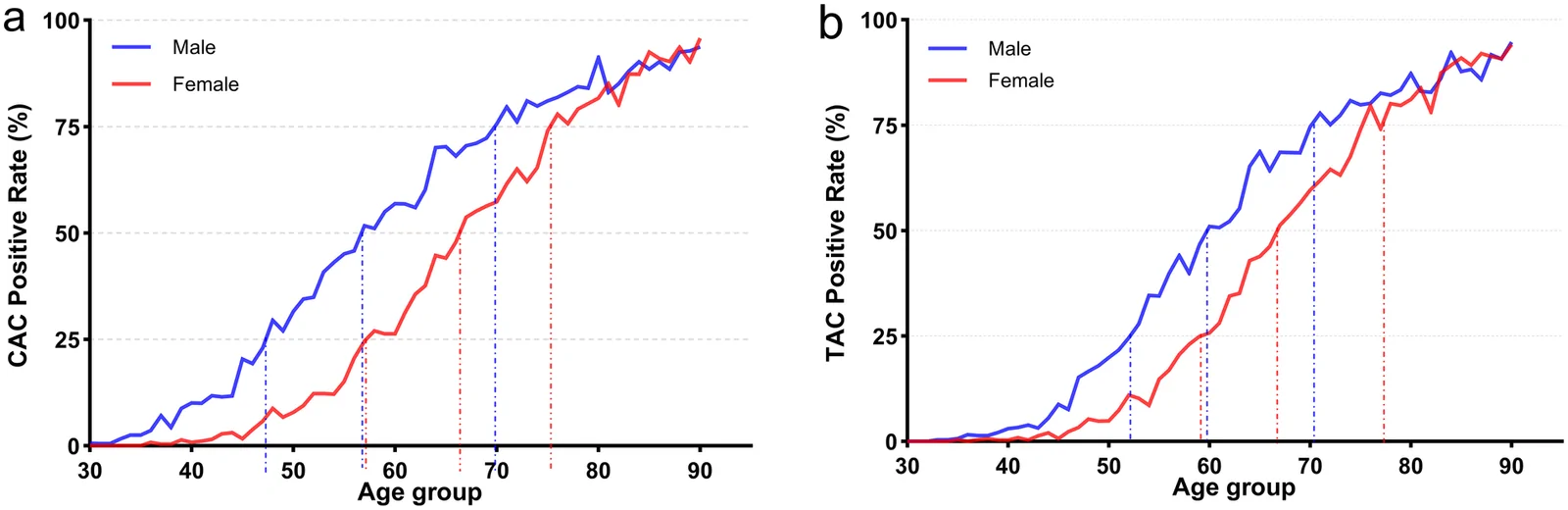
Single-year positivity rate rates and critical age thresholds for coronary artery calcification (CAC) and thoracic aortic calcification (TAC). **(a)** Age-specific trends in CAC positivity rate among men (blue line) and women (red line). The vertical dashed lines indicate the age thresholds at which the CAC positivity rate reached 25%, 50%, and 75% in the respective sex groups. **(b)** Age-specific trends in TAC positivity rate among men (blue line) and women (red line). The vertical dashed lines indicate the age thresholds at which the TAC positivity rate reached 25%, 50%, and 75% in the respective sex groups. CAC, coronary artery calcification; TAC, thoracic aortic calcification.

Notably, women reached each key CAC and TAC prevalence threshold at significantly older ages than men, with longer age lags observed for CAC than TAC (Figure 3).

### Age- and sex-specific distribution of vascular calcification phenotypes

Participants were classified into three mutually exclusive phenotypes: isolated CAC, isolated TAC, and combined calcification. Isolated calcification was rare in men <30 years and women <35 years; linear trend analyses were therefore performed in men ≥30 years and women ≥35 years.

**Figure 4:**
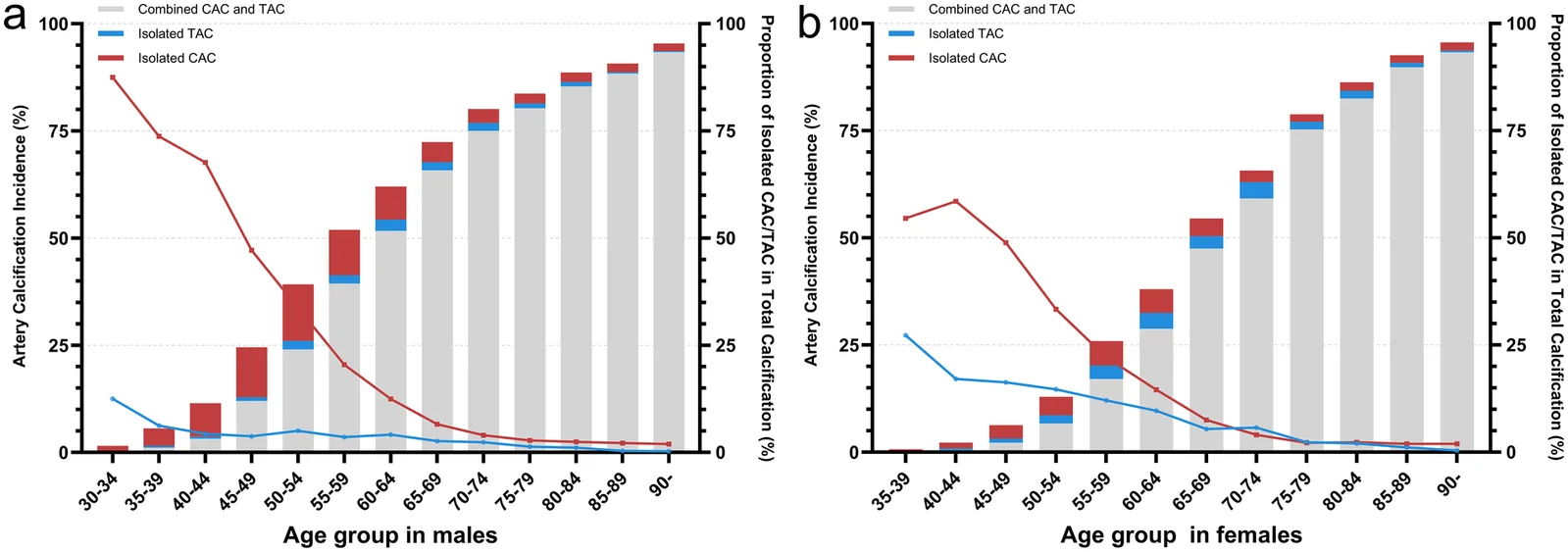
Age-stratified distribution of calcification phenotypes in men and women. **(a)** Distribution of isolated CAC, isolated TAC, and combined calcification in males aged ≥30 years. Linear trend analysis was restricted to this subgroup, while overall positivity rate estimates included the entire male population. **(b)** Distribution of calcification phenotypes in females aged ≥35 years. Linear trend analysis was restricted to this subgroup, while overall positivity rate estimates included the entire female population. No calcification was detected in females aged 30–34 years. All *χ*² tests for age-group differences were significant (all *P* < 0.001). CAC, coronary artery calcification; TAC, thoracic aortic calcification.

The overall prevalence of isolated CAC was 6.3% in men and 2.1% in women (*P* < 0.001). Isolated CAC increased linearly with age in men (*P* < 0.001) but showed no significant linear trend in women (*P* = 0.569). Isolated TAC was more common in women (16.5%) than in men (9.5%, *P* < 0.001) and increased linearly with age in both sexes (both *P* < 0.001). With age, combined calcification gradually became the predominant phenotype. TAC showed a strong linear age association consistent with degenerative change, whereas isolated CAC in women lacked a linear trend, suggesting divergent pathophysiology (Figure 4).

### Clinical stratification by age and sex

Based on the age- and sex-specific distributions of CAC and TAC (Table 2), we categorized participants into three clinically relevant groups by sex: Young (men 0–47 years; women 0–57 years); middle-aged (men 48–69 years; women 58–75 years); elderly (men ≥70 years; women ≥76 years). In the young group, CAC positivity was below 25% in both sexes (5.5% in women, 6.8% in men). In the middle-aged group, CAC positivity ranged from 25% to 75% and increased with age. In the elderly group, CAC positivity exceeded 75% in both women (86.7%) and men (83.7%), indicating a high prevalence of coronary calcification. Given that TAC showed a strong age-dependent degenerative pattern, isolated CAC without TAC may carry unique clinical implications in middle-aged adults.

**Table 2:** Key age thresholds for coronary artery calcification (CAC) and thoracic aortic calcification (TAC) by sex.

Calcification Type	positivity rate Milestone	Male Threshold Age (years)	Female Threshold Age (years)	Male vs. Female Difference (years)
CAC	25%	48	58	10
CAC	50%	57	67	10
CAC	75%	70	76	6
TAC	25%	57	60	3
TAC	50%	60	67	7
TAC	75%	71	78	7

Data are presented as number (percentage). Participants aged 30–89 years were categorized into 5-year age intervals; those aged <30 years were grouped together due to extremely low calcification positivity, and those aged ≥90 years were combined because of near-universal calcification. Sporadic early-onset calcification was identified in individuals <30 years, mostly in those with comorbidities including chronic renal disease, diabetes, hypertension, or malignancy; these rare cases did not alter the overall prevalence pattern. CAC, coronary artery calcification; TAC, thoracic aortic calcification.

## Discussion

The present study shows that opportunistic non-ECG-gated chest CT screening in urban Chinese outpatients identifies numerous young individuals eligible for statin therapy, with consistent applicability for both men and women. For patients younger than the age at which CAC reaches a 25% positivity rate, any visible coronary artery calcium carries clinical significance independent of conventional cardiovascular risk factors. This study boasts multiple strengths: a large cohort free of prior coronary artery disease, 48% female participants, nearly 20% subjects under 40 years old, and roughly 20% participants aged over 70 years. Such a sample size provides sufficient statistical power for age- and sex-stratified analyses. Based on accumulated clinical evidence, international guidelines highlight CAC's pivotal role in primary and secondary ASCVD prevention (2–6). Though CAC and TAC vary in embryonic origin and calcification patterns, both are driven by major traditional cardiovascular risk factors such as aging, hypertension, diabetes mellitus, dyslipidemia and smoking (7, 8). However, the prognostic independence of TAC after adjusting for CAC in remains debated across published work (9), with inconsistent results reported in prior observational studies (10–13). Our results align with the view that CAC signals focal coronary atherosclerosis while TAC mirrors systemic age-related vascular degeneration (7, 14). I solated CAC in young and middle-aged individuals indicates focal, rapidly progressive coronary plaque that precedes systemic atherosclerosis, conferring high specificity for coronary artery disease.

Current mainstream international guidelines do not provide detailed gender-stratified recommendations for coronary artery calcium (CAC) stratification. John et al. reported that adults younger than 50 years with any degree of CAC, were at elevated risk of CVD and death; however, sex-stratified analyses were not performed (15). For adults(men ≥40 or women ≥45 y) with a CAC score ≥75th standardized percentile, the 2026 ACC/AHA guidelines recommend treatment with statin therapy as first line, yet no corresponding guidance is provided for younger populations (1). Our study demonstrates that even a CAC score of 1, corresponding to the 75th percentile, may warrant statin therapy in men aged <47 years and women aged <57 years. This discrepancy is largely attributable to the limited high-quality evidence available for young individuals and women (3). This study enrolled participants aged 1–105 years, with 48.0% being female and a considerable proportion of young subjects, which substantially enriches the existing clinical evidence. The age-related increase in the prevalence of positive CAC and thoracic aortic calcium (TAC) was significantly steeper in men than in women, with the most prominent gender differences observed in middle age. The 25th percentile threshold for CAC positivity in women was approximately 10 years later than that in men, consistent with previous reports (16). Men exhibited a significantly higher prevalence of CAC than TAC across most age groups, indicating earlier onset of coronary atherosclerosis. In contrast, CAC and TAC progressed almost in parallel in women, likely mediated by the vascular protective effects of premenopausal estrogen.

Based on the age- and sex-specific 25th percentile thresholds established in this study, we propose a simplified clinical strategy: in young adults (men<48 years, women<58 years), any detectable CAC should trigger aggressive risk factor modification regardless of concurrent TAC, which aligns with guideline recommendations for individuals with CAC burden in the top 25th percentile (1, 2). This binary approach extends the value of opportunistic CAC screening using routine chest CT and enables early prevention without additional healthcare resources. Notably, self-rated health status is not associated with the presence or severity of CAC (17), suggesting that young individuals with early CAC are often asymptomatic and unaware of their elevated cardiovascular risk. This highlights the importance of such unplanned screening in young individuals at high risk of sudden cardiac death. Major international guidelines, including those from ACC/AHA, recommend CAC testing when eligibility for statin therapy is uncertain (1, 2, 6, 18). Studies have shown that the CAC Agatston score is associated with all-cause mortality in patients with LDL-C ≥ 190 mg/dL (19), and ordinal visual scores of CAC and TAC on chest CT carry similar prognostic value to the Agatston score (20). Nevertheless, these quantitative or ordinal scoring methods remain underutilized in routine clinical practice. The binary visual assessment used in this study is simple and practical, facilitating unplanned screening for asymptomatic high-risk individuals, particularly young adults.

Previous studies demonstrated substantial ethnic and cohort heterogeneity in the prevalence of coronary artery calcium (CAC) among young adults. Western cohorts predominantly consisting of White individuals showed higher atherosclerotic burden overall. Tota-Maharaj et al. investigated a risk factor-enriched cohort with 80% White participants and found that 70% of adults younger than 45 years had a CAC score of 0, and the proportion of CAC-free individuals decreased gradually with age. The CAC Consortium reported an overall CAC prevalence of 34.4% among adults aged 30–49 years (21), and Shaw et al. found that 77.2% of women aged under 55 years had undetectable CAC, a proportion significantly higher than that of age-matched men (16).

East Asian cohorts displayed distinct CAC profiles. Nakanishi et al. reported elevated midlife CAC prevalence rates of 43.1% in men and 26.4% in women (22), which were higher than those observed in our cohort. A recently published Japanese ECG-gated CAC study set the 25% CAC positivity threshold at age 50 for men and age 60 for women; however, CAC scanning served only as a self-paid optional item in physical examinations, which introduced inherent selection bias (23). A national multi-center Chinese CCTA cohort of more than 16,000 symptomatic patients identified a nearly 20-year delay in atherosclerotic onset for females relative to males (24) and a lower overall atherosclerotic burden compared with Western populations. Still, its exclusive enrollment of symptomatic patients receiving contrast-enhanced cardiac CT limited the generalizability to routine outpatient groups.

By contrast, the present study enrolled consecutive outpatients who received routine noncardiac chest CT. It represented an unselected real-world urban Beijing population without artificial risk screening or extra charges for cardiac imaging. It should be noted that the 2026 ACC/AHA guideline includes dual statin indications covering mild CAC of 1–99 AU as well as CAC above age-specific 75th percentile. Consistent with transethnic epidemiological evidence, participants in our Beijing cohort carried markedly lower baseline CAC burden than White populations. Nevertheless, clinicians should cautiously interpret negative CAC findings derived from thin-slice non-ECG-gated chest CT. Even gold-standard ECG-gated coronary CTA dedicated to CAC quantification frequently identifies patients with obstructive coronary lesions despite a CAC score of zero. A large Danish registry cohort by Mortensen et al. (25) demonstrated that 58% of symptomatic patients younger than 40 years with obstructive luminal stenosis had undetectable CAC, whereas only 5% of patients aged ≥70 years presented zero calcium burden. This prominent age difference arises because early atherosclerotic lesions in young individuals are predominantly non-calcified vulnerable plaques invisible to CAC scoring, meaning severe luminal stenosis can exist without measurable coronary calcification. Accordingly, comprehensive cardiovascular risk evaluation integrating traditional risk factors, family history of premature coronary heart disease and clinical manifestations is essential, and risk stratification cannot depend merely on CAC results from routine chest CT alone. According to ACC/AHA guidelines, statin therapy may be safely deferred for 5–10 years among intermediate-risk or selected borderline-risk adults with a CAC score of 0 and no high-risk comorbidities, with repeated risk assessment recommended (5). Similarly, the Canadian Cardiovascular Society (CCS) recommends withholding statin therapy in individuals with a CAC score of 0, with follow-up reassessment within 5 years for those aged over 40 years (26). Moreover, Tota-Maharaj et al. reported that among individuals aged older than 75 years, those with a CAC score of 0 exhibited an excellent prognosis, with a 5.6-year survival rate of 98%, justifying their classification as a very-low-risk group (27). Given the prevalent polypharmacy in this elderly cohort, identifying low-risk individuals with CAC may offer meaningful value for those aged ≥75 years (the concept of de-risking) (28). The present study included approximately 20% of participants aged 70 years and older, representing a sufficiently large elderly cohort. We established a 75% CAC positivity rate as the threshold for defining the elderly subgroup: men aged ≥70 years and women aged ≥76 years. In this age stratum, where the overall CAC positivity rate exceeds 75%, a negative CAC score reliably indicates low cardiovascular risk and favorable long-term outcomes.

This study has several limitations. First, all participants were local residents of Beijing, where life expectancy and medical care standards are among the highest in China. Chest CT examinations were mostly ordered for respiratory complaints or routine health screening rather than cardiac symptoms. With relatively widespread use of chest CT among young urban residents in Beijing, this hospital outpatient cohort has mild referral bias but still well reflects the characteristics of local urban residents. As a result, the proportion of individuals who voluntarily underwent chest CT screening was relatively high. By contrast, the detection rate of asymptomatic individuals through CAC screening would likely be substantially lower in less economically developed regions. Second, variations in climate, diet, lifestyle and cardiovascular risk factors across different geographic regions and ethnic populations lead to heterogeneous CAC prevalence. Thus, our sex-specific age thresholds only apply to urban Beijing residents, and a unified cutoff cannot be generalized to other geographic or ethnic cohorts. Future multi-center studies enrolling diverse populations are needed to develop population-specific CAC screening thresholds. Furthermore, among young individuals, particularly those with a low overall CAC positivity rate, the specific high-risk factors associated with coronary heart disease warrant further investigation. Future prospective studies with long-term clinical outcome follow-up are warranted to externally validate the risk discrimination performance of our age-based CAC thresholds, as our cross-sectional data cannot confirm long-term prognostic benefits of intervention. In summary, this large real-world cohort of urban Beijing outpatients established sex- and age-specific CAC positivity thresholds based on routine non-ECG-gated chest CT. Our data revealed distinct age disparities for detectable coronary calcification between men and women, and highlighted clear differences in vascular calcification progression across White, Japanese and Chinese cohorts. As an opportunistic screening strategy without extra radiation or medical costs, routine chest CT-based CAC assessment identifies young individuals with subclinical atherosclerosis who meet guideline statin criteria, providing practical reference for primary cardiovascular risk screening in urban East Asian populations.

## Data Availability

The raw data supporting the conclusions of this article will be made available by the authors, without undue reservation.
